# Thoraco-Omphalopagus Twins: A Case Report

**DOI:** 10.7759/cureus.87365

**Published:** 2025-07-06

**Authors:** Evgenia Stagaki, Anna Damatopoulou, Marianna Efstratiadou, Nefeli Prompona

**Affiliations:** 1 Obstetrics and Gynaecology, Venizeleio Hospital, Heraklion, GRC

**Keywords:** case report, conjoined twins, siamese twins, thoraco-omphalophagus, twin pregnancy

## Abstract

Conjoined twins are a rare form of congenital disorder in twin pregnancies, associated with significant morbidity and mortality. The terms “conjoined twins” or “Siamese twins” relate to identical twins that are physically connected in utero. In cases where there is a shared heart or other complex anomalies, termination of pregnancy is considered a management option. We report the findings and outcome of a thoraco-omphalopagus twins case.

A 24-year-old woman presented to our hospital’s outpatient department at 13 weeks and two days gestational age. From the first trimester ultrasound, conjoined thoraco-omphalopagus twins were revealed. Counseling was offered including management options and prognosis. Following the couple’s decision this pregnancy was terminated after the patient received pharmacological treatment.

Conjoined twins remain until today a rarely encountered pregnancy; therefore, morbidity and mortality rates of twins are increased. Medical termination of pregnancy can be offered in these cases.

## Introduction

Conjoined twins are a rare form of identical twins, who are physically connected both in utero and after birth. They are always of the same sex, with a higher incidence observed in women (1:3, male to female). The condition occurs in approximately 1:50,000 pregnancies, but due to stillbirths, the overall incidence is reduced to 1:200,000 [1]. The prevalence is higher in regions such as Southeast Asia and Africa. Due to the rarity of this fetal anomaly, management may be challenging for healthcare professionals [1].

The earliest evidence of conjoined twins can be traced back to the Neolithic period, particularly in artistic representations. One of the most notable early depictions is exhibited at the Anatolian Civilisation Museum in Ankara, Turkey, and is the "Double Goddess," a 17 cm marble statue that represents parapagus twins [2]. A similar artifact is exhibited in the San Marco Museum in Florence, Italy, and dates to approximately 80 BC [2]. Throughout history, there has been a significant medical interest in the separation of such twins, with the first recorded attempt to be estimated at around 1100 AD. However, the first successful separation was achieved in 1689 in Basel, Switzerland by Johannes Fatio [3].

Two primary hypotheses, fission and fusion theory, explain the embryogenesis of conjoined twins. The most widely accepted theory is the fission theory, according to which conjoined twins result from incomplete division of the fertilized ovum between 13 and 15 days after fertilization. The second theory, the fusion theory, proposes that conjoined twins result from the early fusion of two distinct embryonic discs [4]. As conjoined twins are always monozygotic, they are typically monoamniotic (sharing a single amniotic sac) and monochorionic (sharing a single placenta). However, fission and fusion theories do not solely contribute to all conjoined twins cases [5].

Conjoined twins can be presented in various forms, with the most frequent type being thoraco-omphalopagus twins accounting for approximately 28% of cases [4]. Classification of conjoined twins is based on the anatomical region where they are joined and the degree of symmetry. In cases of symmetrical twins, the most frequent point of union is ventral (87% of cases), but can also be dorsal (13% of cases) [6]. Ventral symmetrical unions include thoracopagus (19%), omphalopagus (18%), cephalopagus (11%), parapagus (28%), and ischiopagus (11%) twins, with the specific sites of connection being the thorax, abdomen, cranium, pelvis, and variant trunk and lower abdomen/genitourinary system, respectively [2]. Dorsal unions involve craniopagus, rachipagus, and pygopagus twins [2]. On the other hand, asymmetrical conjoined twins, also known as heteropagus parasitic twins, involve an incomplete twin that is attached to another twin at various locations but lacks independent circulation. Classification of conjoined twins can be expanded to describe the number of involved limbs. This includes dibrachius (two upper limbs), tribrachius (three upper limbs), tetrabrachius (four upper limbs), as well as dipus (two lower limbs), tripus (three lower limbs), and tetrapus (four lower limbs) [6].

The diagnosis of conjoined twins is typically made around 7 to 10 weeks of gestation, primarily during first-trimester ultrasound [7]. Early detection is crucial as it enables timely prenatal counseling, an essential step given the high mortality and morbidity rates. If the parents decide to continue the pregnancy, early diagnosis enables delivery planning and ensures appropriate postnatal care, which may include palliative care or, if feasible, surgical separation [7]. Prenatal evaluation during the second trimester generally includes not only routine ultrasound but also echocardiography between 18 to 20 weeks of gestation, because of potential congenital heart disorders that are linked to this anomaly [8]. In addition, magnetic resonance imaging (MRI) can provide more detailed and accurate anatomical information, which helps in the determination of the conjoined type and consequently in the classification. After birth, a postnatal MRI is recommended and should be compared with the prenatal imaging, which can assist in surgical planning. Furthermore, three-dimensional (3D) printing has emerged as a valuable tool in preoperative planning [8].

Early diagnosis and prenatal evaluation are essential for providing appropriate counseling regarding the survival prospects of the fetuses and neonates and the potential for surgical separation [9]. The success of surgical separation largely depends on the shared organs. When vital organs are involved, the risk of death of one or both twins is extremely high. Overall prognosis remains poor as 40-60% of cases result in miscarriage or stillbirth, while only 18% survive beyond the first 24 hours [4]. Consequently, this situation presents ethical dilemmas, and the decision-making process ultimately lies on the wishes and values of the parents [10].

## Case presentation

A 24-year-old gravida two para one woman after a natural conception, presented at our institution at 13 weeks and two days of gestation. A detailed medical history was obtained, revealing a family history of twinning from both parental sides and no other significant findings. Regarding her obstetric history, she had a full-term pregnancy (39 weeks and six days) one year prior, which resulted in a cesarean section due to fetal heart rate abnormalities.

**Figure 1:**
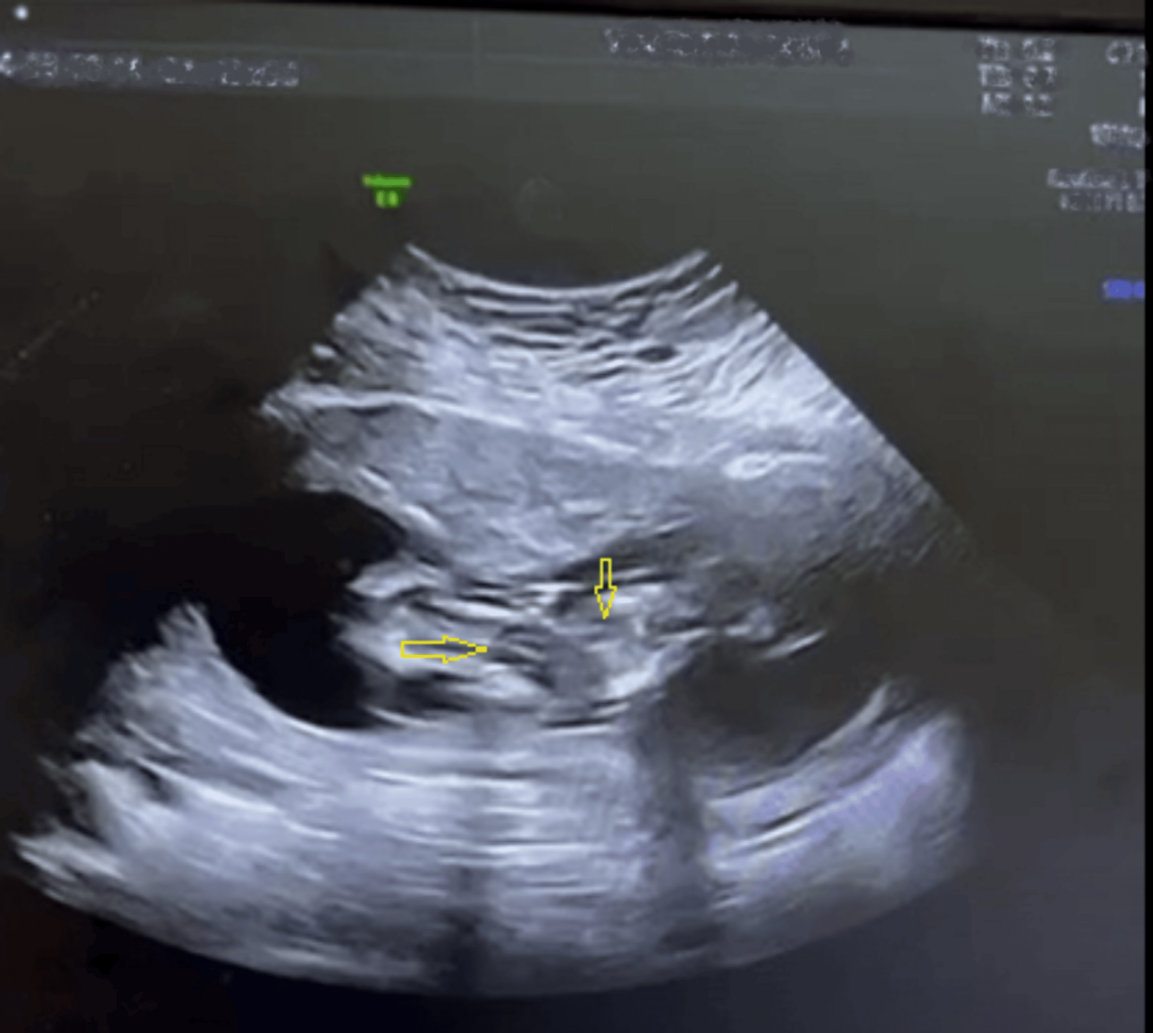
Ultrasound in 13+5 weeks (the yellow arrows show the two distinct hearts)

The first antenatal ultrasound was performed elsewhere at five weeks of pregnancy and confirmed the endometrial sac of gestation and the patient was prescribed folic acid supplementation. A transabdominal ultrasound was conducted at our outpatient department and revealed thoraco-omphalopagus conjoined twins, fused at the thorax and abdomen. The fetuses were of similar size but with significant differences in nuchal translucency measurements. The twins had a single shared placenta and a single liver but had separate stomachs, urinary bladders, and distinct hearts, each with its own heartbeat (Table 1, Figure 1).

**Table 1:** Findings from the ultrasound in 13+2 weeks CRL: crown-rump length; NT: nuchal translucency; bpm: beats per minute Reference ranges: CRL (65–78 mm), NT (≤2.8 mm), heart rate (120–160 bpm)

Fetus	A	B
Findings	Alive	Alive
Cardiac activity	Present	Present
Heart rate	158 bpm	135 bpm
CRL	68.5 mm	69.4 mm
NT	11.00 mm	2.10 mm
Placenta	Posterior low	Posterior low

Following the diagnosis of thoraco-omphalopagus conjoined twins, a detailed counseling session was conducted in which the parents were thoroughly informed of the diagnosis, prognosis, and available management options. An MRI was recommended to provide further evaluation and guide the potential treatment strategy. After careful consideration, the couple decided to terminate the pregnancy without performing an MRI scan. The patient was provided with a prescription for 600 mg of mifepristone, along with detailed instructions.

**Figure 2:**
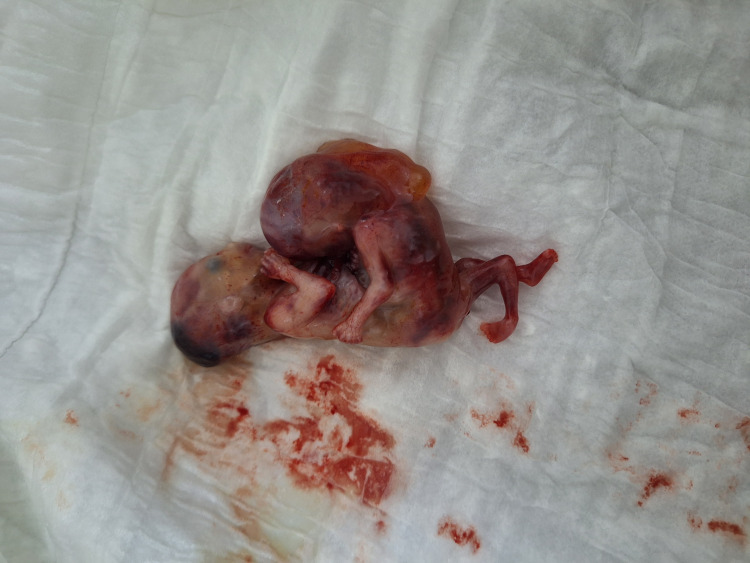
The aborted fetuses

Thirty-six hours after administration of mifepristone, the patient was admitted to the hospital to continue the termination process with 400 mcg of transvaginal misoprostol. Two hours later, the patient delivered both fetuses and the placenta vaginally. After the abortion, the fetuses were subsequently examined, and the findings were consistent with the initial ultrasound diagnosis (Figure 2).

## Discussion

Early diagnosis of conjoined twins is of great importance, due to the impact it has on the lives of the parents. The condition occurs in approximately 1:50,000 pregnancies, but due to stillbirths, the overall incidence is reduced to 1:200,000 [1]. Conjoined twins with fusion of the thorax and upper abdomen (thoraco-omphalopagus) account for 28% of all cases [4]. Immediately after the diagnosis, accurate prenatal counseling must be conducted in a way that respects the couple’s ethical, cultural, and religious beliefs. This is highlighted in another case report of thoraco-omphalopagus conjoined twins, which underscores the importance of early detection and detailed counseling [11]. The counseling team should not only include maternal-fetal medicine specialists and neonatologists but also pediatric surgeons, pediatric anesthesiologists, radiologists, cardiac surgeons, and social workers. The team must discuss extensively all possible options, including the possibility of pregnancy termination, expectant management, and the potential for surgical separation. This statement aligns with the multidisciplinary approach recommended by a reference center in Brazil, which has over two decades of experience [12]. In addition to discussing the prognosis, the team should also highlight the implications of both separation and non-separation on the infants, the nature of surgical separation, and the potential psychosocial and economic impacts. Findings from a single-center study report that 50-70% of parents opt for pregnancy termination [13]. Data deriving from a large retrospective review revealed 27.2% of cases opting for elective pregnancy termination when all cases were included [14]. Considering that elective pregnancy termination may not be permitted globally by law, the rate increases to 50.7% (103 out of 203 cases). In countries where termination is permitted, most couples proceed with this decision [14]. According to similar findings, the decision of pregnancy termination is based on the severity of the deformity and the prognosis of surgical separation [2]. This is in line with our own study indicating the observed trend in parental choices under poor prognosis conditions. Emotional support and counseling are advised before and after decision-making due to potential grief and self-blame that may arise. A significant percentage of couples would value an opportunity to discuss and share their thoughts with parents having a similar experience [15].

Since 1950, over 200 surgical separations of conjoined twins have been performed, with the success rates gradually improving due to advances in early diagnosis, enhanced prenatal monitoring, improved postnatal care, anesthesia development, and postoperative critical care [6]. Developed countries are more likely to benefit from these advancements, since in lower-resource countries, there are still many limitations that have an impact on the postnatal and postoperative care [6]. Surgical separation is typically considered for omphalopagus and pygopagus conjoined twins and in some cases for craniopagus and thoracopagus twins. However, separation is not feasible and therefore not suggested in cases of cephalopagus, parapagus, and rachipagus-conjoined twins. Therefore, the decision regarding pregnancy management is influenced by both regional healthcare status and classification of the conjoined twins [6].

The majority of post-separation deaths occur within the first 24-48 hours. Among the various types of conjoined twins, thoracopagus twins have the highest mortality rate, exceeding 80%, because cardiopulmonary anomalies may arise in most cases. In contrast, omphalopagus conjoined twins have the best outcomes, with a post-separation mortality rate of approximately 20% [6]. Unfortunately, there are no reports describing such operations being performed in our country, Greece. This fact reveals a huge national gap in the medical field. Undoubtedly, this impacts prenatal counseling and the decision-making process, highlighting the need for future international collaborations with leading countries in this field in order to manage such cases and provide training for future generations of medical professionals.

In the present case, the couple’s decision was to terminate the pregnancy after the accurate diagnosis of the conjoined twin pregnancy and extensive counseling due to the poor survival rate, the potential detrimental health effects, and the impact that this would have on the infant’s quality of life as well as on the entire family.

## Conclusions

Despite the medical and surgical evaluation, there are significant challenges in prenatal diagnosis and management of thoraco-omphalopagus conjoined twins. Detailed first-trimester ultrasonography is crucial for early detection and is pivotal in guiding clinical decisions and counseling. The prognosis and potential for surgical separation are determined by the presence of separate vital organs, particularly the heart. Multidisciplinary collaboration is essential to provide comprehensive care and support to affected families. Continuous research will enhance our understanding and improve management strategies for this rare condition.
